# Multi-Level Information Storage Using Engineered Electromechanical Resonances of Piezoelectric Wafers: A Concept Piezoelectric Quick Response (PQR) Code

**DOI:** 10.3390/s20216344

**Published:** 2020-11-06

**Authors:** Christopher Hakoda, Eric S. Davis, Cristian Pantea, Vamshi Krishna Chillara

**Affiliations:** Acoustics and Sensors Team, Materials Physics and Applications (MPA-11), Los Alamos National Laboratory, Los Alamos, NM 87545, USA; chakoda@lanl.gov (C.H.); esdavis@lanl.gov (E.S.D.); pantea@lanl.gov (C.P.)

**Keywords:** information storage, piezoelectrics, electromechanical resonances, Quick Response (QR) code

## Abstract

A piezoelectric-based method for information storage is presented. It involves engineering the polarization profiles of multiple piezoelectric wafers to enhance/suppress specific electromechanical resonances. These enhanced/suppressed resonances can be used to represent multiple frequency-dependent bits, thus enabling multi-level information storage. This multi-level information storage is demonstrated by achieving three information states for a ternary encoding. Using the three information states, we present an approach to encode and decode information from a 2-by-3 array of piezoelectric wafers that we refer to as a concept Piezoelectric Quick Response (PQR) code. The scaling relation between the number of wafers used and the cumulative number of information states that can be achieved with the proposed methodology is briefly discussed. Potential applications of this methodology include tamper-evident devices, embedded product tags in manufacturing/inventory tracking, and additional layers of security with existing information storage technologies.

## 1. Introduction

We propose an information storage methodology that uses an array of electromechanical resonators, namely piezoelectric wafers, to store information in their engineered, frequency-dependent, electromechanical response characteristics. Compared to conventional information storage techniques, the proposed methodology is different in the following ways:

- While traditional methodologies typically use a binary encoding (i.e., a ‘1′ or ‘0′ bit), the frequency-dependent response of a piezoelectric wafer allows for multiple states by utilizing enhanced/suppressed electromechanical resonances. For example, one can achieve ternary encoding with three states ‘0′, ‘1′, and ‘2′.
- The piezoelectric wafers with different information states can be made to look visually identical to each other. Hence, the stored information cannot be decoded using a visual tool such as a camera.
- The methodology does not rely on a line-of-sight access and can also be used in scenarios where piezoelectric wafers can be embedded inside objects or enclosures, thus making it less susceptible to tampering.

With the above advantages, the proposed methodology has strong potential for improved information security when compared to some traditional methods for information storage. However, this methodology is not intended to serve as a replacement for existing techniques such as optical quick response codes [1,2] and radio frequency identification (RFID) tags [3]; instead, it can be used in conjunction with them as an additional layer of security. In addition, while the proposed methodology achieves the aforementioned features at the expense of higher costs, these are justifiable for certain classes of applications (e.g., the storage and transport of sensitive materials) that require non-traditional tamper-proof or tamper-evident technologies [4]. 

The use of piezoelectric materials for information storage is not new; ferroelectrics (a sub-category of piezoelectrics) are used in ferroelectric random access memory (FeRAM) [5,6,7]. While conventional FeRAMs use binary information storage, recent research [8] has shown that FeRAMs are capable of multi-level information storage. This is accomplished by altering the duration of the polarizing electric field, which results in varying degrees of uniform remnant polarization in the ferroelectric thin-film. In contrast to the above approach, the proposed methodology achieves multi-level information storage by engineering the spatial polarization profiles of piezoelectric wafers [9,10] to enhance/suppress specific electromechanical resonances. To the best of our knowledge, such an approach that utilizes the electromechanical resonance characteristics of piezoelectric wafers for information storage has not been attempted in the literature. The only reference we could find that was relevant to the proposed theme is that of information storage in piezoelectric powders [11]. However, the authors of that paper adopted a completely different approach that uses the temporal relaxation characteristics of phonon echoes for information storage and retrieval and is very different from the proposed approach.

We next present our approach for achieving multi-level information storage using piezoelectric wafers.

## 2. Multi-Level Information Storage Using Piezoelectric Wafers

To achieve multiple information states, we use three types of polarization profiles for the piezoelectric wafers:

(1) Depoled: A piezoelectric wafer that is not polarized. When measured, this does not show any electromechanical resonances.
(2) Uniformly Poled: A uniform polarization throughout the piezoelectric wafer with all the dipoles aligned along the thickness. When measured, a wafer with this polarization profile yields all the electromechanical resonances typically observed for the wafer’s geometry.
(3) Non-Uniformly Poled: A polarization profile that is engineered to enhance a specific resonant mode. When measured, a wafer with this polarization profile yields a stronger resonance at the targeted mode while the surrounding modes are suppressed.

These three types of polarizations are used to represent three distinct information states: ‘0′, ‘1′, and ‘2′, respectively. The term ‘trit’ from ternary encoding is used to refer to the three information states. A benefit of altering the polarization profile of a piezoelectric wafer, but not its geometry, is that the wafers representing different ‘trits’ look exactly identical to each other. This provides an additional layer of security and acts as a deterrent for potential tampering from visual imaging methods.

To demonstrate the multi-level information storage, six circular Lead Zirconate Titanate-5A (PZT-5A) wafers are polarized according to the three polarization types mentioned previously—two wafers for each polarization type. All piezoelectric wafers are 0.4 mm-thick, 10 mm in diameter, and manufactured by Boston Piezo-Optics Inc, Bellingham, MA, USA. 

**Figure 1:**
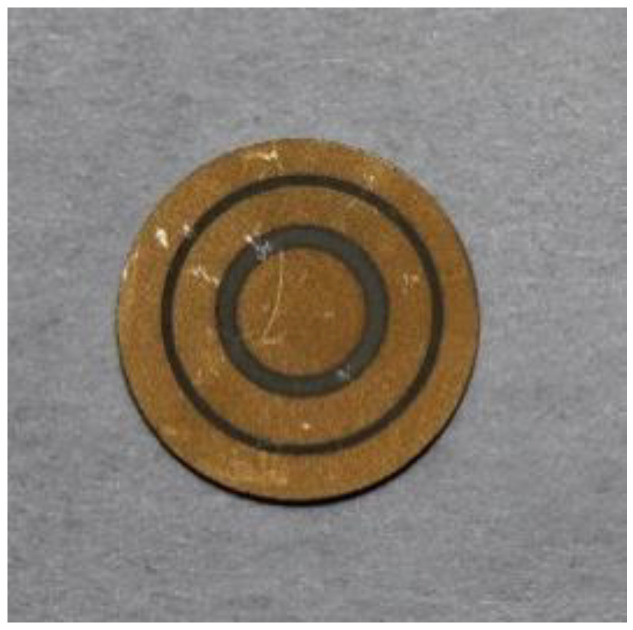
Image of the non-uniformly-poled piezoelectric wafer. The non-uniformly poled PZT-5A wafer is designed to enhance the third radial mode.

The non-uniformly-poled piezoelectric wafer [9,10] is designed to enhance the electromechanical resonance of the third radial mode of vibration. These radial modes [12,13] vibrate in a Bessel-type pattern with annular rings. For a third radial mode, there are three annular rings; hence, the polarization profile of our non-uniformly poled piezoelectric wafer is designed to mimic the three annular rings, as shown in Figure 1. The corresponding dimensions and the poling direction of the annular regions are detailed in Table 1. Note that the wafers are polarized using just the two stable polarization states, up and down, that are spatially distributed in an annular pattern. 

**Figure 2:**
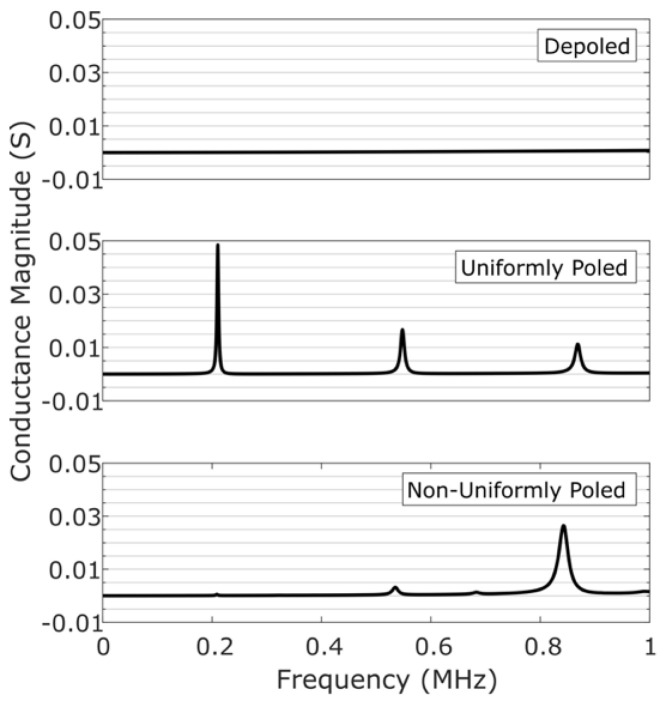
Magnitude of the conductance spectrum of the depoled, uniformly-poled, and non-uniformly poled piezoelectric wafers.

The electromechanical resonances of each piezoelectric wafer are determined by measuring the conductance spectrum with an Omicron Bode 100 vector network analyzer. The magnitude of the conductance spectrum yields clean peaks that can be easily thresholded and are representative of the strength of the electromechanical resonance. Figure 2 shows the conductance spectrum magnitudes for the depoled, uniformly-poled, and the non-uniformly poled piezoelectric wafers. 

In Figure 2, the frequency range is chosen so that only the first three radial modes could be seen. Compared to the spectrum of the uniformly-poled piezoelectric wafer, the spectrum of the non-uniformly-poled piezoelectric wafer shows an enhanced third radial mode, while the first and second radial modes are significantly suppressed. A slight frequency shift and an unexpected resonance between the second and third radial mode are most likely a result of non-optimized spacing between polarized regions in the non-uniformly-poled piezoelectric wafer [14]. The depoled piezoelectric wafer does not show any resonances, as expected.

**Figure 3:**
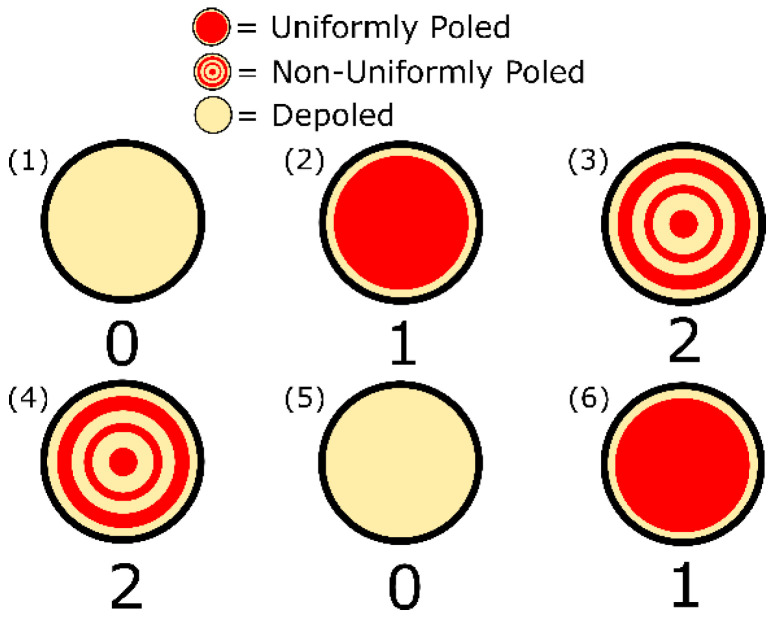
Polarization and arrangement of the piezoelectric circular wafers. The first row reads in ternary encoding as ‘0 1 2,’ and the second row reads as ‘2 0 1.’ The wafers in the 2-by-3 array are numbered in the upper left corner to relate this schematic to the measured conductance spectra shown in Figure 5 and the results in Table 2.

The six piezoelectric wafers are arranged in a 2-by-3 array, as shown in Figure 3. We refer to this array as a concept Piezoelectric Quick Response (PQR) code due to its similarity with the 2D-array of pixels (1′s and 0′s) used in a conventional optical QR code. The first row of the 2-by-3 array consists of the three types of polarizations in the order of the ‘trit’ they represent, while the second row is mixed up to prevent potential bias based on positioning. The piezoelectric wafers are labelled with a number in the upper-left corner, and the intended ‘trit’ is labelled below the corresponding piezoelectric wafer.

**Figure 4:**
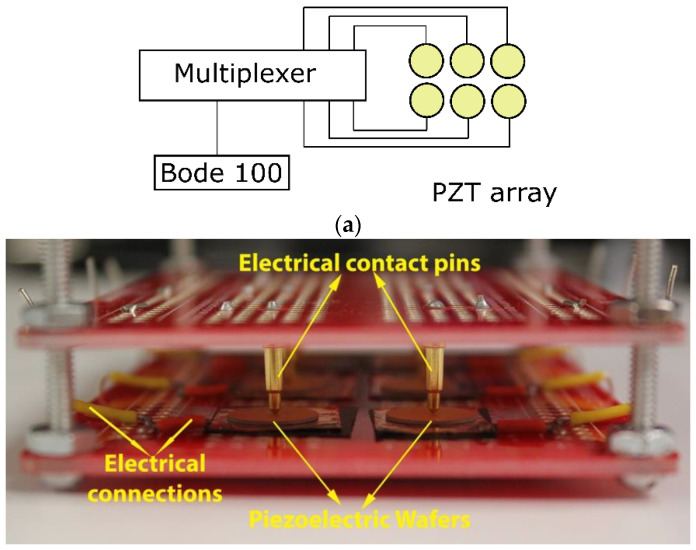
(**a**) Schematic of the experimental setup and (**b**) a picture of the piezoelectric wafer array in the experimental setup. The copper grounding pads and the pogo pins can be seen making contact with the piezoelectric circular wafers.

All six piezoelectric wafers are wired to a Keithley 3706A multiplexer, as shown in Figure 4a schematic. We used Mill-Max Mfg. Corp., spring-loaded pogo pins to make electrical contact with each piezoelectric wafer and a thin copper film for structurally supporting and grounding each piezoelectric wafer. A picture of the complete setup is shown in Figure 4b. While the experimental setup for this proof-of-concept study may seem rudimentary, all the components can be scaled, fabricated, and streamlined for practical deployment. We next discuss information retrieval from the PQR code.

## 3. Information Retrieval from the PQR Code

Information retrieval from the PQR code is accomplished by normalizing the conductance spectra shown in Figure 2. Upon normalization, the following criteria are used for retrieving the encoded data:

- *‘0′ trit*: there are no peaks above threshold at the first and third radial modes.
- *‘1′ trit*: there is a peak above threshold at the first radial mode, but not at the third radial mode.
- *‘2′ trit*: there is a peak above threshold at the third radial mode, but not at the first radial mode.

This information retrieval is made more robust by windowing the data around the first and third radial modes and by identifying the presence/absence of a peak in each window. The window around the first radial mode is chosen to be 0.15–0.25 MHz, and the window around the third radial mode is chosen to be 0.8–0.9 MHz. This accounts for any variability in the measured conductance spectrum resulting from the fabrication process. 

**Figure 5:**
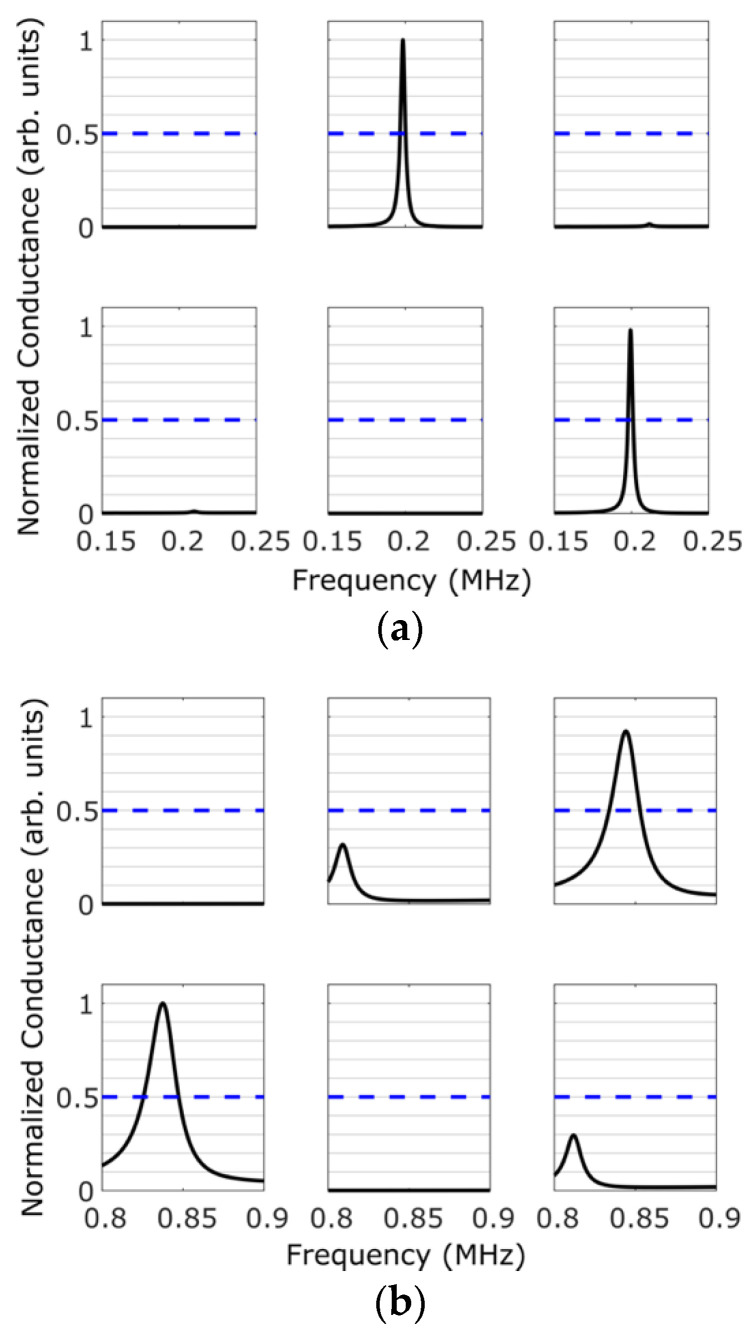
Magnitude of the conductance spectra measured using a multiplexer and a vector network analyzer. The tolerance level chosen for this proof-of-concept is indicated by a blue-dashed line. (**a**) The spectrum corresponding to the window of the first radial mode and (**b**) the spectrum corresponding to the window of third radial mode.

The spectra for the two frequency windows are shown in Figure 5a,b. The blue-dashed line is the threshold chosen for this setup. Figure 5a displays the spectrum within the frequency window corresponding to the first radial mode. Figure 5b displays the spectrum within the second frequency window corresponding to the third radial mode. 

By using the aforementioned criteria for reading the encoded data, we are able to successfully retrieve the intended ternary encoding, as shown in Table 2. This demonstrates the proposed concept of information storage and retrieval from a PQR code. It should be noted that the voltage or the electric field strength used to retrieve the data is much smaller than that used during the polarization of the wafers. This ensures two things: (1) The applied voltage during information retrieval does not alter the information state of the wafer, and (2) it does not heat the sample beyond the Curie temperature so as to depolarize the wafer completely resulting in loss of information. Both these aspects ensure that stored information can be retrieved multiple times without altering the information states of the wafers. In other words, the information state of each wafer is pre-fixed when it is initially polarized and cannot be altered to any other state during information retrieval. Thus, the PQR code cannot be tampered with, a feature that is especially useful for designing tamper-proof tags/seals.

While the above proof-of-concept code was demonstrated with three different information states, the number of information states can be further increased by using polarization profiles that target higher-order resonant modes. For example, if we target the fourth radial mode, we can represent up to four information states and so on until we run out of radial modes. This occurs as the thickness mode of resonance will begin to dominate at higher frequencies. For circular wafers with thickness-to-diameter ratio of <0.05, 6–8 radial modes can be targeted, and one can thus achieve 6–8 distinct information states per wafer. This forms the basis for the PQR code’s multi-level information storage. In general, if one can achieve “N” (an integer) distinct information states using the proposed approach, then a PQR code with “P” (an integer) piezoelectric wafers can be used to store $N^{P}$ cumulative information states. For the PQR code depicted in Figure 3, we have N = 3 and P = 6; hence, it can have $3^{6}=729$ cumulative number of states that are significantly higher compared to that achievable using binary storage that can only have $2^{6}=64$ states. 

While the circular wafers only allow us to target the radial modes, wafers with rectangular geometry would give the freedom to choose from four types of modes [15,16]—in-plane extensional, in-plane shear, and in-plane bending modes along the length and width of the rectangle. Each mode-type has multiple resonances that can be targeted for enhancement/suppression. Thus, the available set of information states grow at least four-fold (4×) compared to what is achievable with circular wafers. Efforts in this direction are underway [16,17]. 

The proposed concept can easily be scaled for practical applications by using wafers of smaller size that are spatially arranged in a high-density, small footprint arrangement. It was shown in reference [8] that the polarization state of a wafer could be controlled at much smaller length-scales than discussed in this article. This enables one to achieve multiple information states using non-uniform polarization in smaller-sized wafers. Even a lower-end estimate of 9 wafers/$in^{2}$ (3-by-3 array) with just a ternary encoding, as presented in this article, could give a total of ~20,000 states/$in^{2}$. Note that these estimates are only based on a simple encoding method presented in this article. More complex encoding schemes can result in a higher number of information states and will be the subject of future investigation. 

Finally, our future work will also investigate the possibility of embedding PQR codes inside of objects or enclosures. In this scenario, if one can interpret the vibrations on the surface resulting from the PQR code inside the object/enclosure, it would then be possible to read the encoded data using a laser Doppler vibrometer (LDV). Since the proposed information storage methodology relies on electromechanical resonance frequencies, even coarser surface scans using LDV or other similar approaches that can determine the spatial distribution of vibrational frequencies on a surface suffice for this application. This will allow for the encoded data to be read from a distance quickly and could serve as an alternative to the proposed method in this article, which requires an electrical contact to work.

## Figures and Tables

**Table 1:** Polarization and dimensions of the non-uniformly poled piezoelectric wafer.

Ring #	Inner Radius (mm)	Outer Radius (mm)	Polarization Rirection
1	0	1.78	Up
2	2.38	3.59	Down
3	3.85	4.98	Up

**Table 2:** The encoded data retrieved from the conductance spectra using the threshold-based criteria.

Piezoelectric Wafer #	(1)	(2)	(3)	(4)	(5)	(6)
Encoded Data	0	1	2	2	0	1
